# Automatic Calibration of Microscopic Traffic Simulation Models Using Artificial Neural Networks

**DOI:** 10.3390/s23218798

**Published:** 2023-10-29

**Authors:** Rodrigo F. Daguano, Leopoldo R. Yoshioka, Marcio L. Netto, Claudio L. Marte, Cassiano A. Isler, Max Mauro Dias Santos, João F. Justo

**Affiliations:** 1 Department of Electronic Systems Engineering, Escola Politécnica da Universidade de São Paulo, São Paulo CEP 05508-010, SP, Brazil; daguano.rod@gmail.com (R.F.D.); leopoldo.yoshioka@usp.br (L.R.Y.); marcio.netto@usp.br (M.L.N.); joao.justo@usp.br (J.F.J.); 2 Department of Transport Engineering, Escola Politécnica da Universidade de São Paulo, São Paulo CEP 05508-010, SP, Brazil; claudio.marte@usp.br (C.L.M.); cassiano.isler@usp.br (C.A.I.); 3 Department of Electronics, Federal University of Technology—Paraná, Ponta Grossa CEP 84017-220, PR, Brazil

**Keywords:** automatic simulation, microsimulation, traffic models, calibration, artificial neural network

## Abstract

Traffic simulations are valuable tools for urban mobility planning and operation, particularly in large cities. Simulation-based microscopic models have enabled traffic engineers to understand local transit and transport behaviors more deeply and manage urban mobility. However, for the simulations to be effective, the transport network and user behavior parameters must be calibrated to mirror real scenarios. In general, calibration is performed manually by traffic engineers who use their knowledge and experience to adjust the parameters of the simulator. Unfortunately, there is still no systematic and automatic process for calibrating traffic simulation networks, although some methods have been proposed in the literature. This study proposes a methodology that facilitates the calibration process, where an artificial neural network (ANN) is trained to learn the behavior of the transport network of interest. The ANN used is the Multi-Layer Perceptron (MLP), trained with back-propagation methods. Based on this learning procedure, the neural network can select the optimized values of the simulation parameters that best mimic the traffic conditions of interest. Experiments considered two microscopic models of traffic and two psychophysical models (Wiedemann 74 and Wiedemann 99). The microscopic traffic models are located in the metropolitan region of São Paulo, Brazil. Moreover, we tested the different configurations of the MLP (layers and numbers of neurons) as well as several variations of the backpropagation training method: Stochastic Gradient Descent (SGD), Adam, Adagrad, Adadelta, Adamax, and Nadam. The results of the experiments show that the proposed methodology is accurate and efficient, leading to calibration with a correlation coefficient greater than 0.8, when the calibrated parameters generate more visible effects on the road network, such as travel times, vehicle counts, and average speeds. For the psychophysical parameters, in the most simplified model (W74), the correlation coefficient was greater than 0.7. The advantage of using ANN for the automatic calibration of simulation parameters is that it allows traffic engineers to carry out comprehensive studies on a large number of future scenarios, such as at different times of the day, as well as on different days of the week and months of the year.

## 1. Introduction

Transport planning and operation have become critical activities worldwide, particularly in urban regions. Before any new intervention in a specific traffic system, engineers usually analyze the impacts of their decisions in future scenarios. These scenarios allow dimensioning solutions that could support practical operation and planning actions to define transport policy strategies. Computational modeling is essential to support these actions, especially when dealing with future scenarios.

A microscopic traffic model characterizes vehicle maneuvers individually based on human driving behavior and provides a complete trajectory, tracking the position of each vehicle from origin to destination. On the other hand, macroscopic traffic models describe traffic flow over a road link using aggregated variables, such as flow (the number of vehicles passing through a road section), speed (the time it takes to cover a given distance), and density (the number of vehicles in a link at a given time). There are intermediate traffic models that fall between these two models. For instance, mesoscopic models calculate some traffic flow elements at a macroscopic level and others at a microscopic level, while submicroscopic models focus on the sub-levels of vehicles and/or drivers, such as the mechanical engine performance of a vehicle or the physiological speed perception of a driver [1].

Macroscopic models for traffic simulations can support long-term planning actions, such as quantifying the impacts of new road infrastructure on a specific area. On the other hand, microscopic traffic simulations aim to model the stochastic interactions observed in transport systems formed by dynamic and complex subsystems, where the aggregated and static models cannot adequately explain the operational and tactical problems observed in traffic engineering. Therefore, traffic microsimulators combine computational resources, such as geo-referenced databases, tools, and algorithms, to represent the urban context, vertical and horizontal signaling systems (dynamic or not), and automata agents governed by behavioral algorithms that define their interactions with any element of the simulated environment.

Microscopic traffic models detail individual behavior, such as lane-changing and car-following maneuvers, and route and departure time choices. For example, Cantisani et al. [2] investigated the effects of user behavior, road design, and flow conditions on initial speeds, acceleration rates, and merging speeds/positions along the acceleration lanes to determine their adequate lengths and guarantee safe merging operations. Giuffrè and Granà [3] used a traffic microsimulation model to estimate the capacity functions in double-lane roundabout entries, whereas Osorio and Nanduri [4] modeled the traffic conditions using information from a macroscopic analytical model, combined with the outputs of a microscopic and emission simulator.

Likewise, the decision-making process for new transportation infrastructure investments and the analysis of road network updates have also benefited from simulation models and related tools [5], which are usually employed to assess and compare multiple scenarios on a what-if or sensitivity analysis [6]. A comprehensive review of the state-of-the-art on simulation models has been presented by Barceló [7].

Despite their advantages in virtually representing real-world traffic conditions and detailing the interactions between vehicle performance and travel behavior under transportation infrastructure interventions, microscopic traffic models require numerous parameters to replicate such conditions adequately [8]. As a result, the simulation tools embedded in these models typically provide average values to those parameters, for example by comparing default values and best-guesses based on optimization procedure [9].

In this sense, the calibration of microscopic simulators essentially consists of determining the values of parameters that provide the most realistic representation of the overall traffic conditions of a scenario, thus minimizing the differences between the outputs of a model and observed field data [10]. However, despite the existing methods for determining the optimal parameter values of microsimulation models, there is still no consensus regarding an adequate and automated procedure to find the microscopic simulation parameters that best represent the traffic conditions of a real-world network.

This paper presents a new method to automatically calibrate the parameters of urban traffic networks by combining microscopic simulation models and artificial neural networks (ANNs). The ANNs used in this project are multi-layer perceptrons (MLPs) trained with back-propagation methods. Different variations have been used (stochastic gradient descent (SDG), Adam, Adagrad, Adadelta, Adamax, and Nadam) to identify their impact on training convergence, with the aim of assessing whether they can learn from some different scenarios (traffic conditions in the same urban site) and then generalize the calibration procedure to help address other scenarios.

In our method, the microscopic traffic model that best fits the observed field data is adjusted by the parameters estimated by the ANNs to match the traffic condition. The ANN was previously trained to exploit a wide range of different configurations, adjusting itself in each case to find the correct match between the parameters and the corresponding results (traffic conditions) of the microsimulation. We applied the proposed approach to two real-world sets of links and nodes of the network in the metropolitan region of São Paulo (Brazil), considering different sets of parameters to be calibrated.

The use of microscopic traffic simulation poses significant challenges in defining the most suitable parameters that replicate user behavior within traffic flow. While various tools and methods have been used to estimate these parameters, there is still no consensus on the most effective approach. Additionally, the potential of artificial neural networks as a supporting tool for these problems remains largely unexplored. Therefore, the contributions of this study are twofold. First, we propose a method to automatically calibrate multiple parameters of microscopic traffic simulators, such as driver behavior and vehicle capability parameters, used in microsimulation models embedded in traffic simulators. Second, we assess the capabilities of ANNs in calibrating microscopic traffic simulations. To our knowledge, there is only one study in the literature that has used ANNs for this purpose [11] in order to calibrate the microscopic traffic simulator VISSIM [12] on roundabout simulations.

The remainder of this paper is organized as follows. A background and literature review of microscopic traffic simulation methods are presented in Section 2, and the proposed methodology is detailed in Section 3. Section 4 describes the experiments used to assess the method, and Section 5 discusses the results of the experiments. Finally, conclusions and rows for future research are presented in Section 6.

## 2. Related Research

Many efficient methods have been proposed in the literature to overcome the challenges in calibrating microscopic simulation models; for instance, real-time and offline calibration using artificial neural networks [11], calibration of origin-destination matrices for large-scale networks [13], and separate or simultaneous behavioral and flow parameter calibration [14].

Punzo et al. [15] provided evidence that a subset of calibrated parameters in microscopic traffic simulation models is sufficient to reproduce actual traffic conditions using simplified car-following models. In addition, Chu et al. [16] stated that small-scale traffic simulations are more affected by driving behavior parameters than more extensive and complex models.

Other research considered sensitivity analyses and trial-and-error methods for calibrating traffic signal systems [17] and lane-changing behavioral decisions [18], notwithstanding their usual time-consuming and resource-intensive characteristics [19]. Sensitivity analysis has been extensively used to select the most influential parameters in microscopic traffic simulations. For example, the one-at-time (OAT) approach has assessed how uncertainty in one parameter affects the model outputs while keeping the other parameters fixed. Despite its effectiveness, such a procedure does not comprise the effect of several parameters simultaneously. Nevertheless, multi-factor analysis of variance (ANOVA) has already been used to analyze the effect of several parameters on a dependent variable to determine their single effect and interactions [20].

In contrast, more sophisticated calibration techniques have also been developed. For example, gradient-based search methods estimate the gradient of the simulation results, which often results in multiple non-convex local minima objective functions of the calibration parameters. The stochastic and highly nonlinear relationships between the parameters and the objective function in these calibration methods usually involve non-closed-form formulations [6]. Despite their ability in dealing with more complex experiments, such methods usually require large-scale datasets and substantial efforts to implement.

Likewise, simulation-based optimization (SO) techniques comprise general-purpose algorithms to address the calibration of the parameters in traffic models. SO algorithms rely on iterative procedures to determine the parameters that best fit the simulation outputs to the observed data [10]. At each iteration, a new set of values was assigned to the parameters based on a given rule, and a predefined error measure was calculated from the difference between the simulation output and field data. The optimization process is completed when the error measure reaches a specific value [21].

The genetic algorithm (GA) is one of the most used SO algorithms in applications to microscopic simulation, for example, using the speed-density relationship [19] and simulating a vehicle decision in intersections [22]. The performance of such a technique has also been tested and improved when combined with the Tabu Search heuristic [23]. Simultaneous perturbation stochastic approximation (SPSA) algorithms [24] and variations, such as weighted SPSA (W-SPSA) [25], have also been extensively used [26]. Other attempts to calibrate traffic simulation models involve multi-start algorithms, Tabu Search, Monte Carlo sampling, artificial neural networks (ANN), and the Extended Kalman Filter (EKF) [10]. These techniques benefit from the current availability of computational infrastructure that allows repetitive tasks to be executed to optimize traffic simulation parameter sets [27].

More recently, metamodels (or surrogate models) have been used to calibrate traffic simulation models by computing the gradient of an analytical and deterministic approximation of an objective function updated using simulation results [6], with applications to network models [28] and microscopic traffic simulation [10]. A review of metamodels was presented by Barton et al. [29]. Osorio and Bierlaire [6] first addressed metamodels for the general-purpose calibration of simulation-based transportation models. More recently, Zhang et al. [28] detailed the procedures for calibrating these models in large-scale traffic simulators and applying them to a single behavioral parameter estimation that controls route choice. Since then, other applications have been presented, such as dynamic transportation optimization [30], origin-destination matrix calibration [31], microscopic car-following models [32], and calibration of agent-based multimodal traffic microsimulation [10]. Despite their effectiveness, the design, implementation, and analysis of such models are not straightforward.

Several works have shown techniques used for calibrating microscopic simulation models while also highlighting the lack of a standardized protocol for this purpose. Chowdhury et al. [33] used a microscopic simulation model to study the impact of road design, traffic, and driver behavior on merging maneuvers. They employed an empirical approach and TransModeler software to analyze video data and estimate the parameters for vehicle classification, critical distance, and gap acceptance. However, the results specifically described traffic within the Italian region. Various tools and methodologies have been employed to accurately simulate urban traffic flow and enable the prediction of the effects of different traffic solutions. For instance, Bieker et al. [34] utilized the commercial software VISSIM, incorporating input data of the road network, traffic demand, traffic lights, and infrastructure, to create three real-world traffic simulation scenarios in Bologna, Italy, and reduce the efforts of users in generating the scenarios. Zambrano-Martinez et al. (2017) used the open-source software SUMO, along with the DFROUTER module and a heuristic approach to generate Origin-Destination matrices utilizing actual data from loop measurements. Their model was applied to Valencia, Spain, and its performance was compared to data from Cologne (Germany) and Bologna (Italy) [35].

Amirjamshidi and Roorda [36] employed a genetic algorithm to optimize a multi-criteria objective function to calibrate microscopic traffic simulation models. The objective function aimed to balance speeds, traffic counts, and acceleration/deceleration patterns, while simultaneously minimizing the Root Mean Square Error between the estimates and field measures. Three different calibrated models were compared, considering the simulated driving cycles and cycle parameters, the estimated parameters, the Vehicle-Specific Power (VSP) distribution, and emission factors. These parameters were then used to compute traffic microsimulation emissions.

Karimi et al. [37] employed traffic data gathered through video surveillance to simulate a real-world highway using VISSIM. They calibrated the parameters of the simulation model using *a posteriori* multi-objective particle swarm optimization (MOPSO) method, aiming to minimize differences between the simulated and observed headway distribution capturing longitudinal movements, as well as the observed and simulated lane changes at various locations, taking into account the lateral maneuvers of vehicles. The calibration process was carried out in a single step. A comparison was performed between this method and other optimization methods, commonly known as *a priori* optimization methods (particle swarm optimization, genetic algorithm, and whale optimization), which involve aggregating objectives into a single function by assigning weighting coefficients to each objective before running the algorithm. The results demonstrated that the *posteriori*-based method yielded more accurate solutions than the other tested algorithms with respect to the objectives. Building on this approach, Karimi and Alecsandru (2019) [38] adopted a similar approach to simultaneously capture the safety and operational aspects of a network, such as the number of conflicts and the time series of speed. The MOPSO method was once again used, leading to better results.

Karimi et al. (2019) [37] used video surveillance data to simulate a real-world highway in VISSIM and calibrate its parameters using a multi-objective particle swarm optimization (MOPSO) method. It aimed to minimize the differences between the simulated headway distribution and the real-world observed headway distribution, as well as the differences between the observed and simulated number of lane changes at different locations. The MOPSO method was compared with other *a priori* optimization methods, such as particle swarm optimization, genetic algorithm, and whale optimization, and it provided more accurate results. Building on this approach, Karimi and Alecsandru (2019) [38] adopted a similar method to simultaneously capture the safety and operational aspects of a network. The MOPSO method was once again used, leading to better results.

Instead of relying on VISSIM’s built-in attributes, such as volume-to-capacity ratios, vehicle delay, and queue lengths, a calibration procedure was proposed to compare real-world saturation flow rates with VISSIM degree of saturation at signalized intersections. The procedure involved comparing the differences between the simulated and field relationships of green time and average headway measurements at the fourth or fifth headway position in a signalized intersection, also known as saturation headway. Time headways were collected from video recordings and compared with the simulated time headways of vehicle trajectories on the queue per signal cycle [39].

Maheshwary et al. [40] proposed a method for calibrating the driving behavior parameters of microscopic simulations using VISSIM for heterogeneous traffic (car, bus, and bike). They explored the commonly used techniques for calibration, including linear regression models, non-linear search algorithms, trial and error, and genetic algorithms, which are mainly used for homogeneous traffic conditions. The proposed method included data collection, network coding, comparison of simulation with default values and field data, identification of parameter ranges, experimental design, and optimization using a genetic algorithm. A case study in Kolkata (India) was presented to calibrate the parameters CC0-CC9 of the Wiedemann model through Latin hypercube design, ANOVA, and linear regression, which showed that the travel time of a particular vehicle class depends on other classes.

A five-step method was proposed to calibrate a microsimulation model in VISSIM/VISWALK for pedestrian crossing time using neural networks [41]. The method was applied in the entry leg of a roundabout in Manfalcone (Italy) to calibrate eight parameters (five related to pedestrians and three linked to vehicular traffic) using a social force model implemented in VISSIM/VISWALK and a feedforward neural network. A database of 100 random combinations of input-output values of the simulation parameters and their corresponding pedestrian crossing times were used to train and validate the estimations with the neural network.

In order to improve the accuracy of crash estimates in microsimulation models, Guo et al. [42] proposed a calibration approach that uses the extreme value theory to link non-crash events to crashes. They first calibrated a VISSIM model to match real-world traffic conditions, such as vehicle delay and arrival type, and then used genetic algorithms to match the extreme value distribution to field-measured conflicts. The approach was applied to two signalized intersections in Canada, being effective in estimating safety measures from simulation.

Chun et al. [43] evaluated the performance of an analytical platform and a microsimulation model using video data captured from a drone. They analyzed vehicle trajectories and operations at a single-lane roundabout. They calibrated the critical headway and follow-up headway distribution using Troutbeck maximum likelihood estimation and the Siegloch method, respectively. The Siegloch method was closely related to the HCM 6 capacity model for roundabouts [44]. They also developed a specific method to calibrate the models that took into account restrictions on sight distance, with results that were more consistent with the field data for both models.

Finally, several methods have been proposed to describe driving behavior. Wu et al. (2019) [45] found that the type of leading vehicle influences longitudinal driving behavior based on naturalistic driving data. Wu et al. [46] modeled the non-humanlike lane-changing behavior of autonomous vehicles using an artificial potential field algorithm to improve humanlike ability. They compared this method with other lane change algorithms in real traffic scenarios, which had a better ability to simulate human driving behavior. In their study, Nassrullah and Yousif [47] created a microsimulation model to assess the effectiveness of various temporary traffic management strategies for highway work zones. They designed car-following, discretionary and mandatory lane change rules, gap acceptance, and narrow lane rules and calibrated the model using data from different locations, traffic flow conditions, lane counts, and section types. The calibrated model was verified by comparing the simulated traffic flow to actual field data.

## 3. Background on Microscopic Traffic Models

Modeling individual vehicles in microscopic traffic simulators involves the representation of several elements, such as input parameters related to travel demand (vehicle flows and route decisions), driving behavior (speed/acceleration, reaction time, desired speed, safety distance, look-ahead distance, lane changing, gap acceptance models, among others), and network topology (maximum speeds, turning movements, link capacity).

Microsimulation tools usually require different parameters to describe the vehicle trajectories. For example, the VISSIM microsimulator [12], developed in the 1970s at the University of Karlsruhe, Germany, is based on Wiedemann psychophysical perception car-following model [48]. This model describes the vehicle characteristics and driving behavior using parameters such as desired speed, acceleration, deceleration, and safe following distance. Moreover, the simulation package AIMSUN [49] considers Gipps’ model and requires the definition of maximum speed, acceleration rate, reaction time, and deceleration rate as input parameters. Finally, the benchmarking of microscopic traffic flow models and related parameters is detailed in the literature [21]. In the following, we describe the Wiedemann model in the 1974 (W74) and 1999 (W99) versions.

### 3.1. Wiedemann 1974 Model

According to Gao [50], the W74 model is formulated as follows.

(1) $$u_{n}\left(t+\Delta t\right)=min\left\{\begin{array}{c} 3.6\cdot {\left(\frac{s_{n}\left(t\right)-Ax}{Bx}\right)}^{2}\\ 3.6\cdot {\left(\frac{s_{n}\left(t\right)-Ax}{Bx\cdot Ex}\right)}^{2} \end{array}\;,\;\;u_{f}\left(t\right)\right\}$$

where $u_{n}\left(t+\Delta t\right)$ is the speed update, and *u_f_* is the spatial traffic stream free-flow speed. *s_n_(t)* is the spacing between the leader (vehicle *n* − *1*) and the follower (vehicle *n*). *Ex* is a calibration parameter. Finally, *Ax* and *Bx* are parameters that comprise the distance *d* between two consecutive vehicles, as follows:

(2) d=Ax+Bx,

where *Ax* is the average standstill distance, and *Bx* is the average safety distance. *Ax* represents the desired average distance between two vehicles when stopped in line, which can be expressed as

(3) $$Ax=L+Ax_{-add}+Ax_{-mult}\cdot rndl\left(I\right)$$

where *L* is the leader vehicle length, *Ax_-add_* is the additive factor of *Ax*, representing the minimum value between two successive vehicles in a queue, *Ax_−mult_* is the multiplicative factor of *Ax*, and *rndl(I)* is the normal distribution random variable (between 0 and 1), and *I* is the seed value.

The safety distance *Bx* corresponds to the time required for the leading vehicle to reach its position. The more careful the driver is, the greater *Bx* is. It can be expressed as:

(4) $$Bx=\left(L+Bx_{-add}+Bx_{mult}\cdot rndl\left(I\right)\right)\cdot \sqrt{v}\;L$$

where *Bx_-add_* is the additive factor of *Bx*, *Bx_−mult_* is the multiplicative factor of *Bx*, *v* is the leader’s vehicle speed [m/s], and *rndl(I)* is a value between 0 and 1, which is typically distributed around 0.5 with a standard deviation of 0.15.

### 3.2. Wiedemann 99 Model

The W99 model is similar to the W74 one, with new parameters added to better model the freeway traffic conditions. According to Gao [50], the W99 model is formulated as

(5) $$u_{n}\left(t+\Delta t\right)=min\left\{\begin{array}{c} u_{n}\left(t\right)+3.6\cdot \left(CC8+\frac{CC8+CC9}{80}u_{n}\left(t\right)\right)\Delta t\\ 3.6\cdot {\left(\frac{s_{n}\left(t\right)-CC0-L_{n-1}}{u_{n}\left(t\right)}\right)}^{2} \end{array}\;\;,\;\;u_{f}\left(t\right)\right\}$$

where *CC*0 is the standstill distance [m], *CC*8 is the standstill acceleration [*m*/*s*^2^], and *CC*9 is the desired acceleration [*m*/*s*^2^] at a speed of 80 km/h. In addition to *CC*0, *CC*8*,* and *CC*9 which appear in Equation (5), there are additional adjustable parameters for W99.

The parameters *CC*0 and *CC*1 of W99 were correlated to the parameters of the previous distances *Ax* and *Bx* described in the W74 model. For example, the standstill distance *CC*0 is expressed as follows:

(6) CC0=Ax−L

where *Ax* is the average standstill distance (Equation (3)) and *L* is the length of the leader vehicle.

The parameter *CC*1 is the headway time, i.e., the interval between two consecutive vehicles, where lower values represent more aggressive drivers. The parameter *CC*2 is the car-following distance variation. It restrains the longitudinal oscillation of a vehicle with the vehicle in front. *CC*2 defines the variation between the minimum distance that the vehicle must remain from the pursued vehicle as

(7) CC2=SDx−Bx

where *SDx* denotes the maximum distance at which the vehicle is still considered to be in pursuit.

The parameter *CC*3 is the threshold for entering “Following”. It defines when the deceleration process begins before reaching the safety distance, *Bx*. The parameter *CC*4 is the negative “following” the threshold that controls the negative speed difference concerning the pursued vehicle. Values with lower modules define a driver as more sensitive to changes in the speed of the leading vehicle.

(8) $$CC4=-\frac{\Delta x-SDx}{CC3}-SDV$$

where ∆*x* is the distance between the pursuing vehicle and the pursued vehicle; SDV is the difference in speeds as a function of the distance between the vehicles, defining the threshold from which the following vehicle notices the vehicle ahead.

The parameter *CC*5 is the positive “following” threshold that controls the positive speed difference with the pursued vehicle. It can be interpreted as the previous parameter *CC*4. Values with lower modules define a driver as more sensitive to changes in the speed of the leading vehicle. The parameter *CC*6 represents the speed dependence of the oscillation. It is defined as the influence of distance on speed oscillation. A zero value indicates that the speed oscillation is independent of distance. The speed oscillation started to increase at higher values as the distance increased. The parameter *CC*7 is the acceleration oscillation and defines the actual acceleration of the vehicle during the oscillation process. The parameter *CC*8 is the standstill acceleration. It represents the desired acceleration when starting from a stationary state. Finally, *CC*9 is the desired acceleration at 80 km/h.

## 4. Methodology

An adjustment of the parameters is required for a microsimulation model to reproduce the traffic conditions of the network of interest. These calibrations result in vehicle demand data or driver behavior parameters, assuming that the characteristics of the road network (capacity and signaling systems) mimic real-world conditions. Here, we consider the calibration of vehicle demand data and the driving behavior parameters for the Wiedemann models (W74 and W99) implemented in the microscopic traffic simulator VISSIM [12]. The simulation tool is widely employed by researchers and practitioners to replicate the behavior of drivers and other road users. This tool can model the movements of every vehicle within the traffic flow, including decisions related to car following and lane changing. For this study, we utilized the 10th version of this software (VISSIM 10).

**Figure 1:**
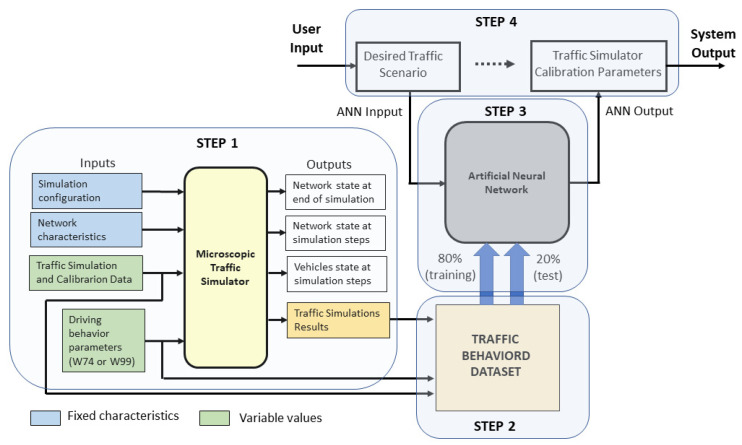
Architecture diagram of the proposed Automatic Calibration of Traffic Microsimulator.

Figure 1 shows the proposed method, using a traffic microsimulator to build an extensive database referring to the input data required to run the simulations, including the set of given values for the parameters to be calibrated and the corresponding output data expressed by measures of performance, such as travel times and flows in the links of the network. Next, the corresponding input and output data generated from the microsimulations are used as input datasets to train a neural network and model a causal relationship between the performance measures of a traffic scenario and the related parameters. Finally, in the decision-making process, the analyst uses traffic data (travel times and flows in the links) as input data to the trained neural network and retrieves related parameters that would provide the most likely simulations representing the observed scenario.

As shown in Figure 1, the method comprises four steps: (a) data generation from microsimulations, (b) data processing, (c) ANN training and testing, and (d) use of the trained ANN to obtain the traffic simulator calibration parameter.

### 4.1. STEP 1: Data Generation

We used a traffic microsimulation to generate a dataset comprising the input data to run the simulations, including the values of the parameters to be calibrated and the output data given by the corresponding performance measures. First, the microstimulator environment is configured with the road network characteristics (link capacities and signaling systems), the travel demand referring to the traffic flows entering the network, movements in the intersections, and values of the behavioral driving parameters of the car-following and lane-changing models. Then, the proposed method can calibrate the driving behavioral parameters or demand flows between origin and destination pairs. In this step, the variables to be calibrated are defined, and their values are established in advance within predefined ranges.

The generation of sets with different values for the vehicle flow demands and driving behavioral parameters was performed repeatedly to build a dataset of instances to be run in the microsimulator. This dataset must be sufficiently large to cover a range of values for calibrating variables. The following section presents simulations performed to obtain the corresponding output data regarding traffic performance measures, such as travel times, speeds, delays, and vehicle counts in the network links. The number of runs and the duration of each simulation depend on the characteristics of the network and its attributes.

The resulting datasets consisted of pairs of input and output data from the microsimulation that were used to train the ANN. The outputs (i.e., the traffic performance measures) and the inputs in the microsimulation are defined as the input data of the ANN. The values of the parameters to be calibrated are the desired outputs of the ANN. Therefore, the ANN provides the most likely values of the parameters that represent the observed traffic conditions used as input data.

### 4.2. STEP 2: Data Processing

This step aimed to filter the dataset obtained from the simulations to train and test the ANN. Specific traffic conditions were observed in the simulations of various scenarios from the previously generated database; in some cases, scenarios with unrealistic outputs may occur owing to road capacity constraints, traffic control specificities, or driving behavior specifications. Those scenarios could hamper the training process of the neural networks and should be excluded from the dataset to be used in the next step of the proposed method. In the experiments described in the following sections, these conditions were observed in less than 3% of the simulations generated in the previous step.

### 4.3. STEP 3: ANN Training and Testing

This step was applied to train and test the ANNs used for pattern recognition. Specifically, a correlation was obtained between the parameters of the traffic simulation model and the corresponding traffic performance measures. The ANN training process of the proposed method comprises four elements: (1) defining the number of epochs, (2) selecting the ANN optimization algorithm, (3) defining the network topology, and (4) selecting an adequate ANN among the tested and trained configurations. TensorFlow libraries [51] and the Keras environment [52] were used to build, train, and test the neural networks.

This study considered ANNs with different numbers of inner layers and neurons. We tested six optimization algorithms to train the ANN topology in each experiment: stochastic gradient descent (SGD) [53], Adagrad [54], Adadelta [55], Adam [56], Adamax [57], and Nadam [58]. We used the mean squared error (MSE) implemented in Keras to test the convergence of the algorithms and the average Pearson correlation between the ANN responses (i.e., the parameters to be calibrated) and the values of the parameters used as input data in the microsimulation runs. We normalized the neural network input data and established a proportion between the training and testing datasets.

The number of epochs must be defined to avoid ANN underfitting and overfitting and to minimize the training error. The Keras environment implements an early stopping procedure, and several training algorithms are comparable regarding the epoch number required for training each neural network. Therefore, this study estimates an adequate number of epochs per optimizing algorithm in each experiment.

Finally, the ideal ANN was built based on the best correlation of its responses compared to the microsimulator parameters to be calibrated and used as input data in the microsimulations. Once the neural networks were trained and tested with the proper topology, several epochs and optimization algorithms were selected.

### 4.4. STEP 4: Deployment of the Traning ANN

From the trained ANN, the user can obtain the best values for the set of parameters of the desired microsimulation scenario by providing practical or hypothetical traffic measures as input data for the ANN.

## 5. Experimental Setup

**Figure 2:**
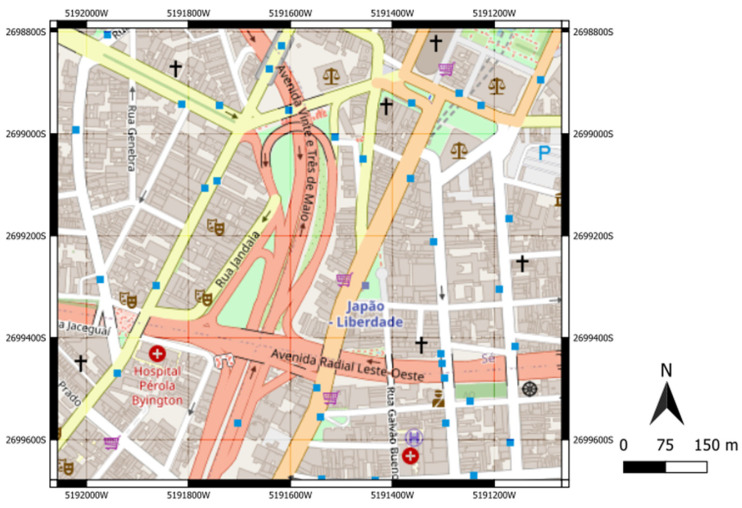
Interchange between Avenue 23 de Maio and Radial Leste in São Paulo (Brazil) [57].

**Figure 3:**
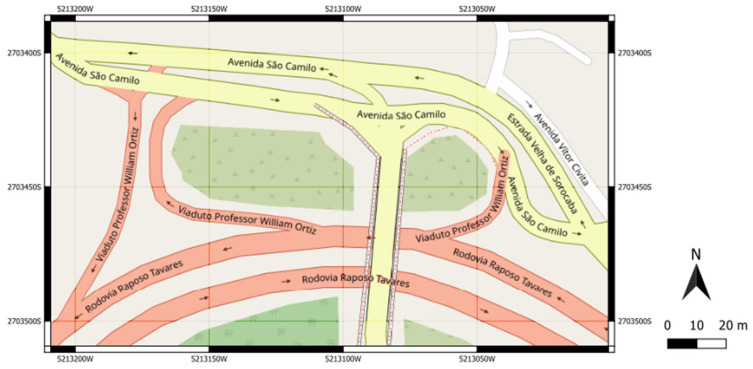
Interchange between Avenue São Camilo and Highway Raposo Tavares in São Paulo (Brazil) [57].

Four experiments involving two urban road interchanges in the metropolitan region of São Paulo (Brazil) were conducted to assess the proposed method. The first interchange (Figure 2) is the intersection between two major expressways in São Paulo, the north-south corridor (Avenue 23 de Maio) and the east corridor (Radial Leste). The second interchange (Figure 3) is the intersection between an expressway that connects a residential area in the northwestern region of São Paulo (Avenue São Camilo) to the highway (Highway Raposo Tavares) that passes through the western region of São Paulo.

### 5.1. E1: Experiment Setup 1

**Figure 4:**
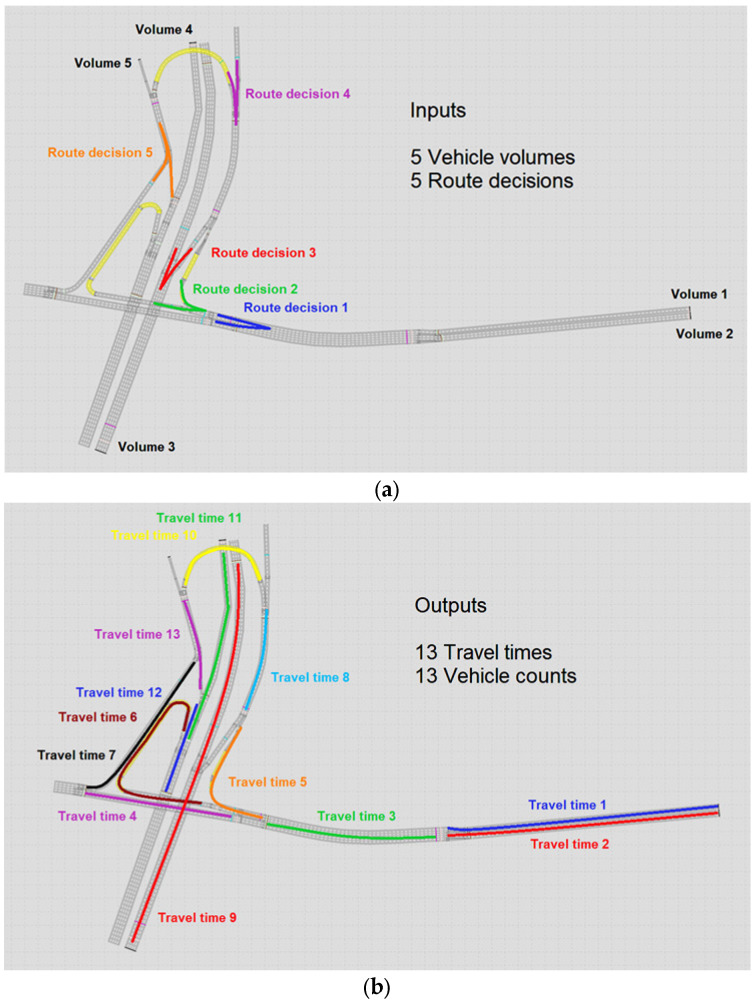
Location of flows and route decisions (**a**) and travel times and vehicle counts (**b**) in the interchange between Avenue 23 de Maio and Radial Leste used in Experiment 1 (E1).

In this experiment, the interchange between Avenue 23 de Maio and Radial Leste (Figure 2) was implemented in VISSIM and used to calibrate the vehicle volumes entering the network and the turning rate percentages at each intersection of the network (henceforth called route decision). The parameters to be calibrated in Experiment 1 (E1) refer to five vehicle volumes randomly drawn from uniform probability distributions between zero and 1800 vehicles per hour per lane and five route decisions randomly drawn from uniform probability distributions between zero and one. The simulation output data used as input data to train and test the ANN comprised 13 travel times and vehicle counts. Figure 4 illustrates the vehicle entrances and turnings, and the links in which the vehicle counts and the travel times were obtained from the simulations.

### 5.2. E2: Experiment Setup 2

**Figure 5:**
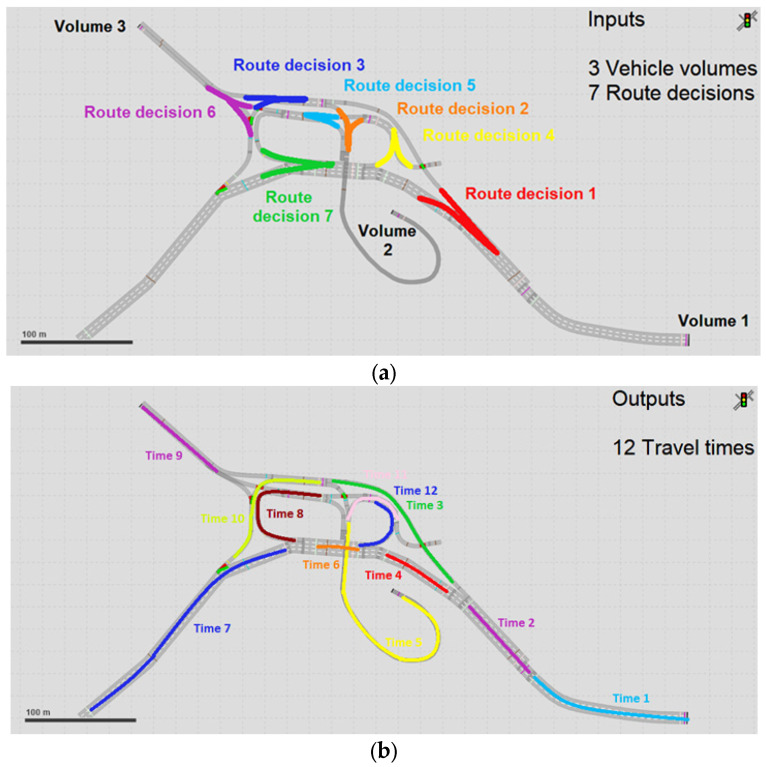
Location of flows and route decisions (**a**) travel times and vehicle counts (**b**) vehicle counts and speeds in the interchange between Avenue São Camilo and Highway Raposo Tavares used in Experiments E2, E3, and E4.

This experiment followed the same sequence as E1, in which vehicle volumes in the entrances of the network and routing decisions were calibrated using the simulated vehicle counts and travel times in specific links as input data to the ANN. In addition, it was executed in the interchange between Avenue São Camilo and Highway Raposo Tavares (Figure 3). In this experiment, three vehicle volumes were randomly drawn from a uniform probability distribution between zero and 1800 vehicles per hour per lane. Seven route decisions following a uniform distribution between zero and one were calibrated considering 12 travel times, 14 vehicle counts, and 14 harmonic average speeds in the links, shown in Figure 5.

### 5.3. E3: Experiment Setup 3

This experiment was also conducted in the interchange between Avenue São Camilo and Highway Raposo Tavares (Figure 3). However, the vehicle volumes and route decisions were established from the data collected in the field. The driving behavior parameters of the Wiedemann 74 (W74) car-following and lane-changing models implemented in VISSIM were calibrated.

More specifically, the input data of the simulations that have been used as output data to the ANN refer to the car-following model behavior parameters of the W74 model:

- Ax, randomly drawn from a uniform probability distribution between 0.5 and 3 m;
- Bx_add, randomly drawn from a uniform probability distribution between 0.5 and 4.0 m;
- Bx_mult, randomly drawn from a uniform probability distribution between 0.5 and 6 m, and the minimum headway distance (MinHdwy) from the vehicle in an adjacent lane as a threshold for the lane-changing maneuver, randomly drawn from a uniform probability distribution between 0.5 and 7.0 m.

The remaining car and driver behavior parameters of the W74 model were fixed to their default values.

### 5.4. E4: Experiment Setup 4

This experiment is analogous to E3, as it considers the same fixed vehicle volumes at the entrances and route decisions in the network shown in Figure 5. However, we replaced the W74 model with the W99 model, which comprises ten parameters (*CC*0–*CC*9) related to acceleration, braking, reaction times, and safe distances. Punzo et al. [15] pointed out the low effectiveness of calibrating all the parameters of a microsimulation at the expense of simplicity, particularly in cases where they do not influence traffic simulations. Therefore, we selected nine of the ten parameters of the W99 model for calibration in this experiment. Only *CC*1 was not analyzed from Model W99 because it is closely correlated with the safety distance (Bx) already explored in the W74 model. For simplicity, in this experiment, the parameters *CC*1 and MinHdwy have been fixed to the VISSIM default values of 0.9 s and 0.5 m, respectively.

These choices and the respective values are randomly drawn from uniform probability distributions based on the interval of the values suggested by Sukennik and Kautzsch [59] as follows according to Table 1:

### 5.5. Experiments Setup Values

These experiments were executed with the following setup values:

- Number of simulations:
  4000 (E1 and E2), 3000 (E3), and 2000 (E4);
- Simulation duration:
  3600 s (E1), 1800 s (E2, E3, and E4);
- Warm-up times:
  600 s (E1) and 300 s (E2, E3, and E4);
- Desired speeds:
  limited to the regulatory speed limit of the roads;
- ANN training/testing dataset ratio was 80/20, and the validation split within the training dataset was 20%;
- Vehicle flows have been converted to equivalent passenger vehicles assuming that motorcycles correspond to 0.5, and buses and trucks are equivalent to two vehicles.

## 6. Results and Discussions

First, the ANN input dataset was generated from the VISSIM microsimulations by applying different values to the parameters to be calibrated and their related outputs, given by the travel times and volumes in the network links. Next, the optimal number of epochs of the ANN, one network for each experiment, was determined considering a fixed network topology of two hidden layers with 50 neurons each and varying Keras optimizing algorithms. Finally, the optimal ANN topology was identified among the different combinations of layers and neurons per layer, given the best-performing algorithm in the previous step. The tested networks comprised structures with 1, 2, and 3 layers, and 2, 3, 5, 10, 15, 20, 30, and 50 neurons, respectively.

In all experiments, the analyses were performed using the Pearson correlation coefficient. This coefficient was calculated between the VISSIM input volumes and ANN outputs. As input to the ANN, travel time and flow values were observed in different stretches of the network, which are the outputs of VISSIM. Moreover, as the output of the ANN, we sought to estimate the probable input volumes of VISSIM in order to generate the desired travel times and flows.

### 6.1. R1: Results of Experiment 1

In Experiment 1, we built an ANN input dataset with 4000 microsimulations running in the transport network, shown in Figure 4, to calibrate vehicle volumes and route decisions.

**Figure 6:**
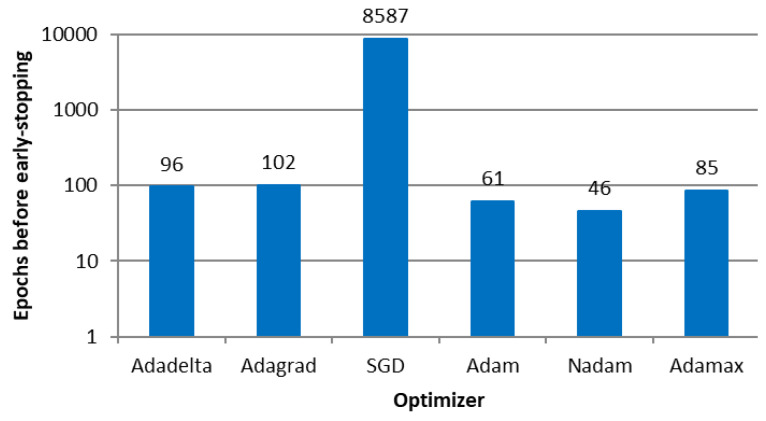
Number of epochs before early stopping callback in E1.

Figure 6 shows the number of epochs before the early stopping callback of each algorithm (i.e., before overfitting the models). Due to the number of input and output variables, the topology of 2 hidden layers with 50 neurons each (the notation is a 50-50 configuration) was fixed, as an initial attempt. To identify the most appropriate ANN topology, we varied the number of hidden layers and neurons per layer, given the number of epochs, as shown in Figure 6.

The SDG optimizer that implemented fixed-sized steps presented more epochs than the others that implemented adaptive-sized steps.

**Figure 7:**
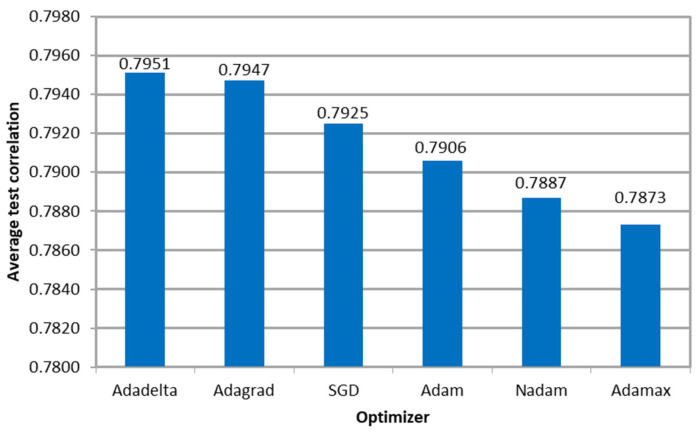
Correlation between the ANN outputs and the parameters used as microsimulation inputs per optimizer in E1.

Figure 7 shows the average Pearson correlation between the microsimulation inputs to be calibrated and the ANN outputs per optimizer, indicating that the models resulted in similar values. Furthermore, the variation in the performance measure among optimizers was less than 1%, indicating that the algorithm’s effectiveness was not affected regardless of the variation in the required number of epochs to convergence.

**Figure 8:**
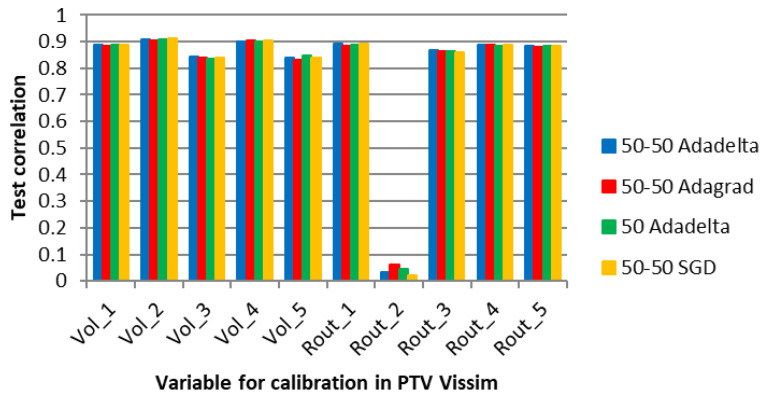
Correlation between the ANN output and the microsimulation input for specific optimizers and network topologies in E1.

Figure 8 shows the four algorithms with the best performance in the average correlation between the microsimulation inputs and the ANN outputs (Adadelta, Adagrad, and SGD with two hidden layers and 50 neurons each, and Adadelta with one hidden layer and 50 neurons).

Figure 8 shows high correlations between the values of the parameters for all the optimizers, except for the route decision “Rout_2”. The volume variables (Vol) did not present significantly higher correlations than the route decision variables (Rout) because the ANN of Experiment 1 did not adjust the vehicle volume entries with only the counts in the edge segments, aiming to reduce the error of the inner segments.

According to Punzo, Montanino, and Ciuffo [15], it is possible to determine a subset of variables that are sufficient for calibration by sensitivity analysis. They suggest that variables that make a small contribution to the output can be ignored. In the case of Rout_2, it is possible that the variable was not captured by the ANN learning process due to complexity limitations.

After the development of the ANN, the individual correlations between variables were estimated. In this initial experiment, the user can review the metrics and decide whether to use the recommendations of the ANN as calibration values. If necessary, variables such as Rout_2 can be manually calibrated or assigned a default value. Alternatively, if the traffic engineer’s requirements are met, the variable may be ignored altogether.

### 6.2. R2: Results of Experiment 2

**Figure 9:**
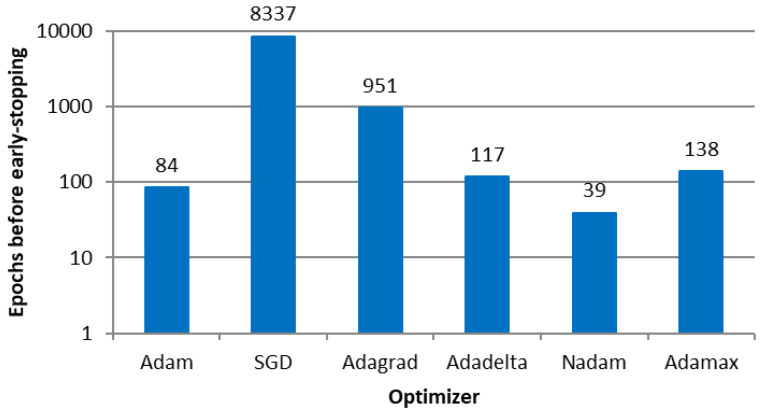
Number of epochs before early stopping callback in E2.

Experiment 2 refers to the calibration of vehicle volume and route decisions in the transport network depicted in Figure 5. We also ran 4000 simulations in VISSIM to obtain a dataset to train and test the neural network. For a fixed neural network with two hidden layers and 50 neurons each, the number of epochs before the early stopping callback, considering the six optimizers implemented in Keras, is shown in Figure 9. SDG provided the highest number of epochs to avoid overfitting analogously to E1, probably due to the specificity of the road network, followed by Adagrad.

**Figure 10:**
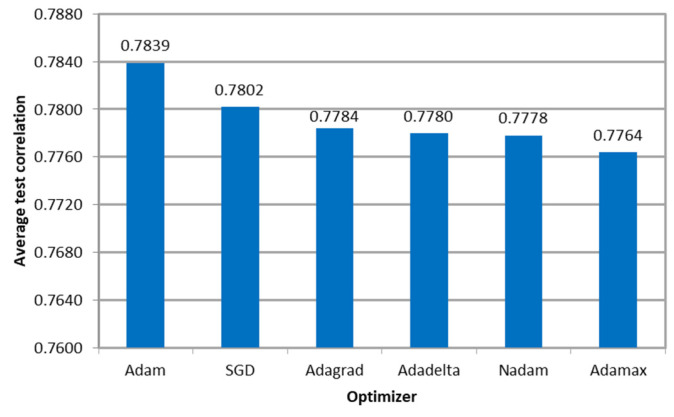
Correlation between the ANN outputs and the parameters used as microsimulation inputs per optimizer in E2.

The average correlation between the ANN output and microsimulation input is shown in Figure 10, where the performance measure does not vary significantly among optimizers, similar to E1.

**Figure 11:**
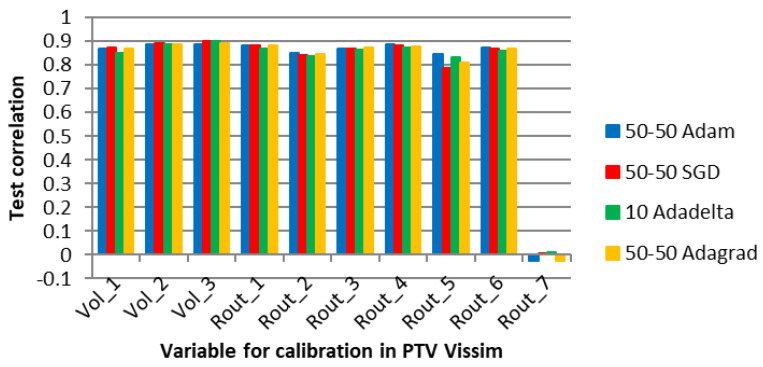
Correlation between the ANN output and the microsimulation input for specific optimizers and network topologies in E2.

Finally, Figure 11 shows the average correlation between the parameters calibrated in the microsimulation and those obtained from the ANN. The best neural network in this experiment had the 50-50 configuration with the Adam optimizer. The correlation varied positively above 0.8 (maximum 0.89) except for the route decision variable “Rout_7”.

According to the E1 justification, the connections between this variable and the PTV VISSIM metrics might be too intricate for the ANN to comprehend while undergoing training. Additionally, it is plausible that the impact of this variable on the metrics is insignificant and not worth considering for calibration, as discussed by Punzo, Montanino, and Ciuffo [15].

Once the ANN development is complete, the user can access the specific metrics of the calibration variables. A traffic engineer has the option to manually calibrate this variable separately, or choose to disregard it and use a default value if the overall calibration results meet the practical requirements of the project.

### 6.3. R3: Results of Experiment 3

**Figure 12:**
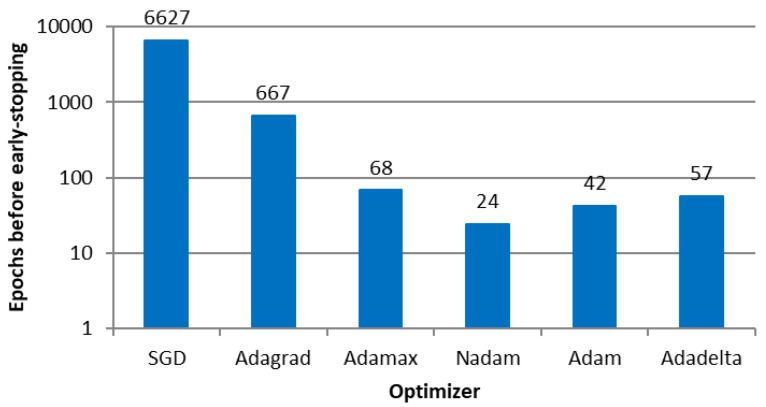
Number of epochs before early stopping callback in E3.

Experiment 3 focused on the same transportation network as E2, which is shown in Figure 5. However, it concentrated on calibrating the W74 car-following and lane-changing parameters. To select four relevant variables related to the Wiedemann 74 car-following model and the lane-change model that influence the aggressiveness of overtaking cars, Fransson’s [60] study was used as a reference. After running 3000 simulations, the number of epochs required by each of the six tested ANN optimizers before early stopping was obtained as shown in Figure 12, where the SDG again resulted in the highest values, followed by Adagrad, similar to previous experiments.

**Figure 13:**
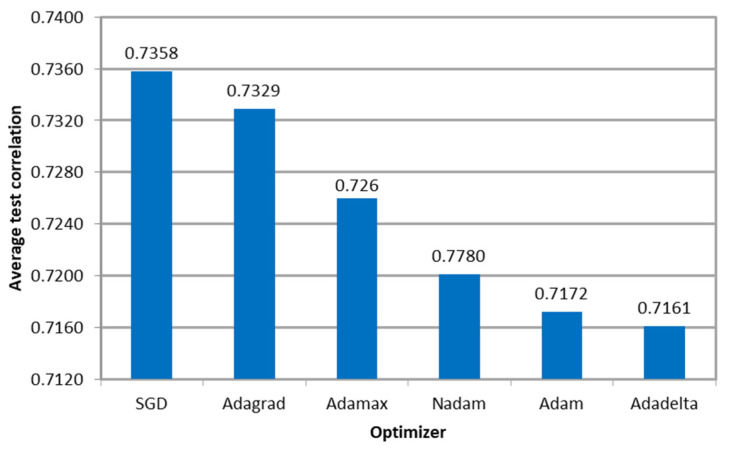
Correlation between the ANN outputs and the parameters used as microsimulation inputs per optimizer in E3.

Furthermore, Figure 13 shows that the average correlation between the microsimulation inputs and ANN outputs varied between 0.7358 (SDG) and 0.7161 (Adadelta). These values are lower than the ones obtained in previous experiments (E1 and E2), as we are not calibrating physical parameters, such as volumes and route decisions, but psychophysical parameters, which are related to the Wiedemann 74 Model (W74).

**Figure 14:**
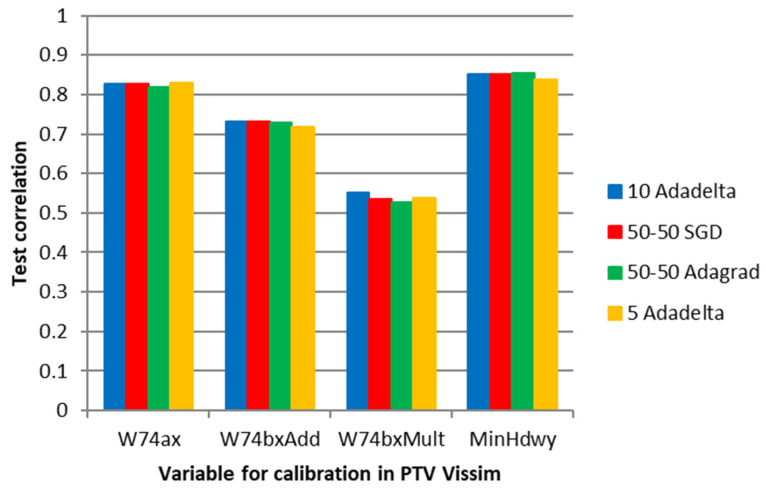
Correlation between the ANN output and the microsimulation input for specific optimizers and network topologies in E3.

Finally, Figure 14 shows that the average correlation between the microsimulation and ANN values is positive for all parameters, with the lowest values referring to W74bxMult.

The high level of abstraction of the driving behavior models, particularly W74, poses a greater challenge for the ANN to achieve optimal calibration performance compared to variables that have more observable effects on the road network, such as travel times, vehicle counts, and average speeds. By following the proposed methodology, a traffic engineer may opt to disregard the W74 variables or use the ANN estimate as a foundation for additional fine-tuning.

### 6.4. R4: Results of Experiment 4

Experiment 4 shares similarities with E2 and E3 as it examines the transportation network shown in Figure 5, but focuses on calibrating the Wiedemann 99 (W99) car-following parameters. The calibration of these parameters is a topic of significant interest for research and discussion, mainly because of their abstract meanings and potential use in modeling the behavior of autonomous vehicles, as suggested by previous studies [59].

**Figure 15:**
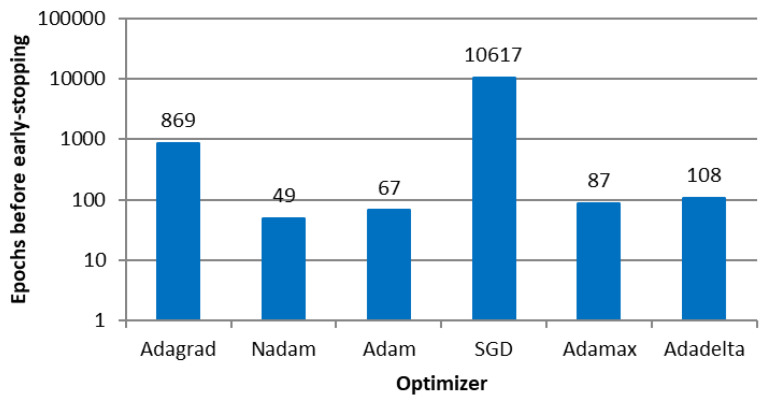
Number of epochs before early stopping callback in E4.

**Figure 16:**
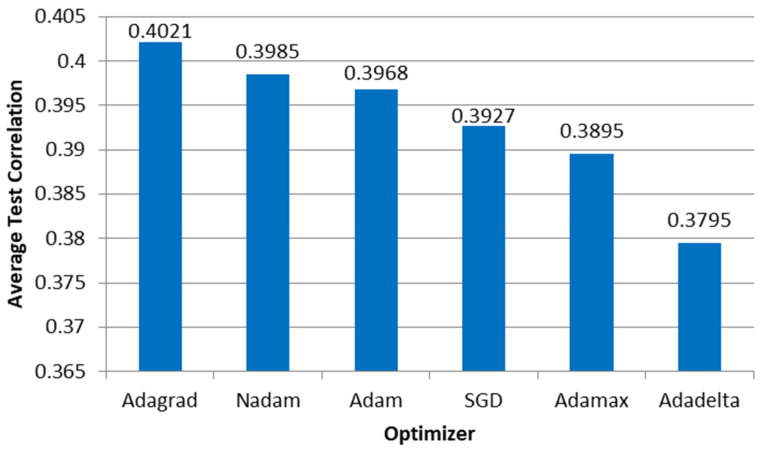
Correlation between the ANN outputs and the parameters used as microsimulation inputs per optimizer in E4.

Figure 15 shows that the SDG and Adagrad require the highest number of epochs before the ANN implements the early stopping procedure at 2000 simulations, the latter significantly higher than the former. In contrast, Figure 16 shows that the average correlation between the microsimulation inputs and the ANN outputs varies between 0.4071 and 0.3795, indicating that the ANN cannot provide accurate values for these kinds of parameters.

**Figure 17:**
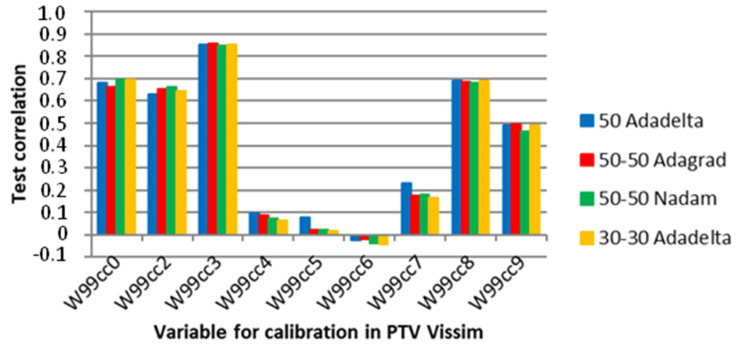
Correlation between the ANN output and the microsimulation input for specific optimizers and network topologies in E4.

Figure 17 shows the average correlation of the parameters in the microsimulation and ANN outputs of the selected network topologies and optimizers. These findings indicate that relying solely on ANN for automatic calibration may not always produce accurate results. Previous studies have shown that calibrating the Wiedemann 99 parameters typically involves straight road segments, roundabouts, or small road networks with low complexity. It is plausible that the mathematical complexity of the relationships between these inputs and the simulator output metrics is too high for the ANN to model accurately, especially given the highly stochastic nature of traffic microsimulations and the abstract nature of the Wiedemann 99 car-following model.

Ultimately, it is essential to obtain feedback from the user to evaluate the effectiveness of the calibration. Given that calibration is frequently an iterative and manual process; the ANN estimates can provide useful starting points for refinement and reduce the workload. As noted by Punzo, Montanino, and Ciuffo [15], it may be possible to only calibrate the W99 parameters that resemble the Wiedemann 74 model since the simulations may be more sensitive to those parameters. This strategy can help streamline the calibration process.

## 7. Conclusions

While various methods and techniques are discussed in the literature, calibrating traffic microsimulation models remains a challenging task for both researchers and practitioners. This work presents a new calibration method for microsimulation models using artificial neural networks. The steps of the proposed method include running several microsimulations with different values for the parameters to be calibrated, collecting and processing the microsimulation output (travel times and link flows), training and testing the ANN configured with a specific number of epochs, layers, and optimizing algorithms, using the microsimulation outputs as input data to the neural networks. For the trained and tested ANNs, it was possible to estimate an adequate set of values for the microsimulation model from the observed traffic measures as input data to the artificial neural network.

To assess the performance of the proposed method in the four experiments implemented in VISSIM, we used the travel time and flows in the links of different networks in the São Paulo metropolitan area. The first and second experiments considered two different networks to calibrate the route decisions, that is, the turning rate percentages at each intersection of the network and the vehicle volumes entering the network. In contrast, the third and fourth experiments calibrated the car-following and lane-changing parameters of the Wiedemann 74 and 99 models in the urban road network in the second experiment. In addition, we estimated the optimal number of epochs before the early stopping of several ANN-optimizing algorithms and compared the correlation between the ANN outputs and parameters used as microsimulation inputs per optimizer and neural network topology.

The results show that the trained and tested ANNs can provide a high correlation between the parameters to be calibrated and obtained as outputs from the ANN and the values used as input data for the microscopic traffic simulation models. More specifically, the correlation between such values was higher than 0.8 for most parameters in Experiments 1 and 2, where the traffic parameters which have more visible effects on the road network, such as travel times, vehicle counts, and average speeds, were calibrated. For Experiment 3, the correlation was lower, around 0.7, due to the attempt to calibrate the psychophysical parameters of Model W74 related to the safety distance. We showed in Experiment 4 that the ANN had more difficulty to achieve high correlation values for psychophysical model, due to the number of W99 parameters being larger than W74.

Our main contribution was to demonstrate that a traffic simulation micromodel can be automatically calibrated with satisfactory accuracy. For this, we proposed to use an ANN trained with a database representative of the traffic demands, to estimate the calibration parameters of the micromodel. The experiments carried out in this investigation showed significant correlations between the estimations of the neural networks and the input data to the traffic simulations in the experiments carried out at different interchanges of an urban road network. Moreover, the results of the proposed method should be of interest to practitioners since, once the neural network is trained and tested, it can be used to obtain calibrated parameters from field data directly retrieved from road sensors (such as cameras and inductive loops) and mobile apps.

We emphasize that the advantage of using ANN for the automatic calibration of simulation parameters is to allow traffic engineers to carry out comprehensive studies on a large number of future scenarios. For example, if it is necessary to evaluate the behavior of the same micromodel during different times of the day, different days of the week, or different months of the year, a great effort would be required to calibrate the micromodel. This is because the traffic demand tends to vary substantially between peak and off-peak hours, as well as between weekdays and weekends, and between typical holiday months and other regular months.

In this work, we use an MLP architecture for the ANN, as it is a well-established technique in many applications. Once the process has been validated, other ANN architectures can be explored in future work, maintaining this approach as an aid to the calibration process, both for micromodels and for network models. For the latter, the ANN can support the estimation of origin-destination matrices and, still working with micromodels, traffic light controls can be incorporated into them. In both cases, new ANN architectures can be explored, keeping the focus on supporting the calibration of simulation models. Additionally, we consider that studies related to aspects of the road network, such as intersections, number of lanes, and road geometry, in addition to psychophysical behavior in the face of anomalous situations, such as rain, road works, and events, such as concerts and sports games, are promising for future research.

## Figures and Tables

**Table 1:** The W99 model parameters and reference value [59].

Parameter	Range	Reference Value
*CC*0 (m)	[0.50, 2.00]	1.5
*CC*2 (m)	[0.00, 5.00]	4.0
*CC*3 (s)	[−12.00, −4.00]	−8.0
*CC*4 (m/s)	[−0.40, 0.00]	−0.35
*CC*5 (m/s)	[0.00, 0.40]	−0.35
*CC*6	[0.00, 12.00]	11.44
*CC*7 (m/s^2^)	[0.00, 3.00]	N/A
*CC*8 (m/s^2^)	[0.00, 5.00]	N/A
*CC*9 (m/s^2^)	[0.00, 3.00]	N/A

## Data Availability

Not applicable.
